# S‐adenosylmethionine and nicotinamide riboside therapy in Arts syndrome: A case report and literature review

**DOI:** 10.1002/jmd2.12395

**Published:** 2023-09-01

**Authors:** Angela Lee, Renatta Knox, Margaret Reynolds, Erin McRoy, Hoanh Nguyen

**Affiliations:** ^1^ Department of Pediatrics, Division of Genetics and Genomic Medicine Washington University Saint Louis Missouri USA; ^2^ Department of Pediatrics and Neurology Washington University Saint Louis Missouri USA; ^3^ Departments of Pediatrics, Division of Ophthalmology Washington University Saint Louis Missouri USA

**Keywords:** Arts syndrome, nicotinamide riboside, phosphoribosylpyrophosphate, PRPP, *PRPS1*, S‐adenosylmethionine

## Abstract

Phospho‐ribosyl‐pyrophosphate synthetase 1 (PRPS1) deficiency is secondary to loss of function variants in *PRPS1*. This enzyme generates phospho‐ribosyl‐pyrophosphate (PRPP), which is utilized in the synthesis of purines, nicotinamide adenine dinucleotide (NAD), and NAD phosphate (NADP), among other metabolic pathways. Arts syndrome, or severe PRPS1 deficiency, is an X‐linked condition characterized by congenital sensorineural hearing loss, optic atrophy, developmental delays, ataxia, hypotonia, and recurrent infections that can cause progressive clinical decline, often resulting in death before 5 years of age. Supplementation of the purine and NAD pathways outside of PRPP‐dependent reactions is a logical approach and has been reported in a handful of patients, two with S‐adenosylmethionine (SAMe) and one with SAMe and nicotinamide riboside (NR). We present the clinical course of a fourth Arts syndrome patient who was started on therapy and review previously reported patients. All patients had stability or improvement of symptoms, suggesting that SAMe and NR can be a treatment option in Arts syndrome, though further studies are warranted.

**Synopsis**

Arts syndrome patients appear to have clinical improvement with S‐adenosylmethionine and nicotinamide riboside; however, larger studies are needed.

## 1 INTRODUCTION

*PRPS1* (OMIM *311850) is located at Xq22.3 and encodes for phospho‐ribosyl‐pyrophosphate synthetase 1 (PRPS1). Phospho‐ribosyl‐pyrophosphate (PRPP) is utilized in the synthesis of purines, pyrimidines, nicotinamide adenine dinucleotide (NAD), and NAD phosphate (NADP), as well as amino acids histidine and tryptophan.^1^ Severe *PRPS1* deficiency, also known as Arts syndrome (OMIM #301835), is an X‐linked condition characterized by congenital sensorineural hearing loss, optic atrophy, developmental delays, ataxia, hypotonia, and recurrent infections. Infections often precipitate worsening of symptoms, and many individuals pass away in early childhood.^2^, ^3^, ^4^ This was first described in 1993 by Arts et al. and to date there have been less than 25 cases reported in the literature.^2^, ^3^, ^4^, ^5^, ^6^, ^7^, ^8^, ^9^, ^10^ Moderate to mild PRPS1 deficiency is associated with hearing loss with later onset ataxia and optic neuropathy, also known as Charcot Marie Tooth Disease X linked 5 (CMTX5, OMIM #311070), or isolated hearing loss, also known as deafness X‐linked 1 (DFNX1, OMIM # 304500), respectively. The incidence of hearing loss related to *PRPS1* is not well characterized. X‐linked hearing loss accounts for <1%–2% of nonsyndromic hearing loss overall and *PRPS1* variants were not identified in large cohorts of patients with nonsyndromic hearing loss.^11^, ^12^ Female carriers of pathogenic *PRPS1* variants range from asymptomatic to varying degrees of isolated hearing loss to a more severe CMTX5 or Arts syndrome phenotype, with significant phenotypic variability.^8^, ^13^, ^14^, ^15^, ^16^ DFNX1, CMTX5, and Arts syndrome were previously distinct entities that are now seen as a continuum related to residual enzyme activity.^8^ There are also *PRPS1* variants associated with PRPS1 superactivity (OMIM #300661). It has been hypothesized that the effect of *PRPS1* variants on the local structure, ATP binding site, allosteric sites, and interface areas can be associated with certain phenotypes; however, this is not well delineated.^1^, ^15^

**FIGURE 1:**
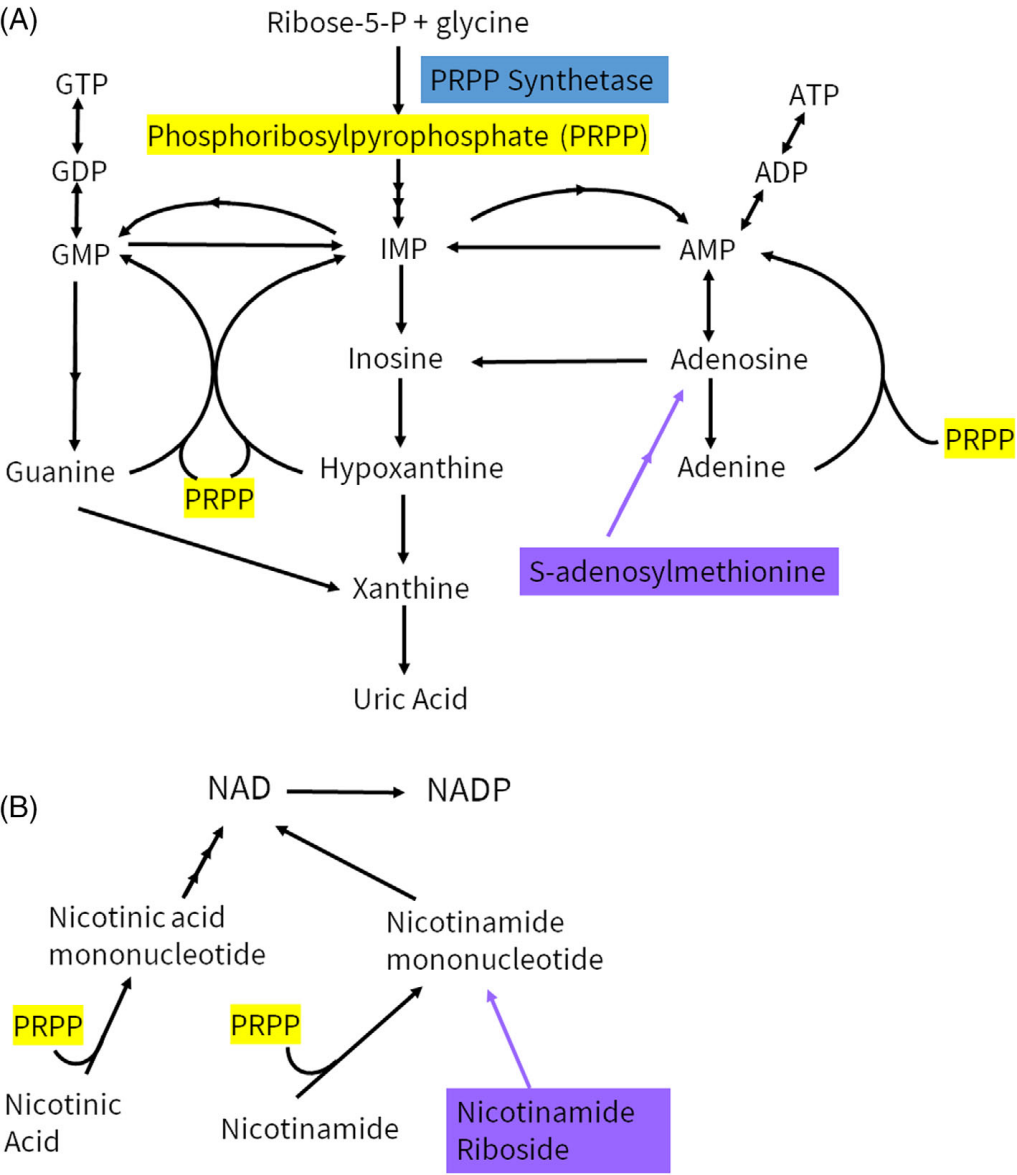
Simplified diagram of the purine synthesis (A) and nicotinamide adenine dinucleotide (NAD) synthesis pathways (B). PRPP (highlighted in yellow) plays a significant role in both pathways. PRPS1 encodes the enzyme PRPP synthetase (highlighted in blue). S‐adenosylmethionine and nicotinamide riboside supplementation is noted in purple.

Given the importance of PRPP in the synthesis of purines and NAD(P), supplementation of these pathways is a logical approach for these patients. S‐adenosylmethionine (SAMe) serves as source of purine precursors that is PRPP‐independent (Figure 1). Similarly, nicotinamide riboside (NR) allows for NAD(P) formation independent of PRPP (Figure 1). Both SAMe and NR have been used for a variety of medical indications, with minimal side effects.^17^, ^18^, ^19^, ^20^ Two Arts syndrome patients were previously supplemented with SAMe, starting in mid‐childhood, with improvement in infection severity and frequency, as well as stabilization of other symptoms.^5^ Another Arts syndrome patient was supplemented with SAMe and NR, starting in early childhood, with improvement in infection frequency and developmental gains.^7^ Here, we present the clinical course of a 28‐month‐old male patient with Arts syndrome, who was started on SAMe and NR supplementation and compare to prior Arts syndrome patients who received SAMe with or without NR.

## 2 CASE PRESENTATION

The patient is the first son of nonconsanguineous white parents. He was born at 36 weeks to a 30‐year‐old G4P3 mother whose pregnancy was complicated by intrauterine growth restriction. His birth weight was 1.89 kg (WHO 0.1st percentile), birth length was 46 cm (WHO 1.4th percentile), and birth head circumference was 31 cm (WHO 0.3rd percentile). He did not pass his newborn hearing screening and was noted to have congenital nystagmus and hypotonia. He was diagnosed with severe congenital bilateral sensorineural hearing loss by auditory brainstem response at 2 months of age and subsequently received hearing aids. An electroretinogram showed retinal dystrophy. Subsequent genetic testing revealed a likely pathogenic variant in *PRPS1* c.383A>T (p.Asp128Val) (NM_002764.4). This specific variant met ACMG criteria PM2, PP3, PP2, PP1, and PP5 with no population frequency in gnomAD, deleterious in silico predictions at a highly conserved amino acid, missense variant in a gene with low rate of benign missense variation (*o*/*e* = 0.08, *Z* = 3.74), co‐segregation in affected family members, and likely pathologic classification in Clinvar and in Mercati et al.^16^, ^21^ His uric acid, xanthine, and hypoxanthine levels were in the low range of normal. His brain MRI was normal, and he underwent cochlear implants at 12 months of age. By 18 months, he was pulling to stand, crawling, and able to sit unsupported with some ataxia. He was babbling but had no words or signs. He did not have a history of recurrent infections; however, family reports relative isolation due to the Covid‐19 pandemic.

After discussion with family, he was started on 200 mg daily (20 mg/kg/day) of SAMe at 19 months, which was increased to 400 mg daily (approximately 40 mg/kg/day) at 20 months. At 20 months, he was also started on 300 mg daily (approximately 30 mg/kg/day) of NR. Medication doses were based on prior dosing and the nearest available capsule size. Parents reported subjective improvement in strength and endurance with therapy. Over the next few months, he made significant developmental gains, including walking with a walker at 21 months. He had done well with occasional upper respiratory tract infections without regression in skills, worsening hypotonia, or increased respiratory needs.

At 24 months of age, he had significant regression after cardiac arrest secondary to respiratory failure from rhinovirus/enterovirus. Full immunologic evaluation—including lymphocyte subpopulations, T and B cell subset distributions, and immunoglobulins—was normal. He had appropriate vaccine titers for vaccines received, as he was under‐vaccinated. Head CT did not show significant changes; however, there was artifact present from cochlear implants. Brain MRI was unable to be obtained due to incompatibility with cochlear implants. At discharge 3 weeks later, he had lost head control, was not able to sit unsupported, roll over, or feed by mouth, prompting G‐tube placement. He was discharged with cough assist and vest to assist with secretion clearance, but did not require other respiratory support. Over the next couple of months, he regained many milestones and acquired additional milestones. At 26 months, he was sitting independently, pulling to stand, crawling, speaking (one to two words), and has resumed eating by mouth. He has had another upper respiratory tract infection without any clinical decline. Uric acid and purine metabolites remained similar while on SAMe and NR. Methionine remained in the normal range.

The patient's mother, who had childhood onset hearing loss, was found to be a carrier for the *PRPS1* c.383A>T variant. Other female family members, including a maternal aunt with hearing loss, are undergoing cascade genetic testing.

## 3 DISCUSSION

To date, three other Arts syndrome patients have been previously treated with SAMe (*n* = 2) or SAMe and NR (*n* = 1). Their clinical data is summarized with our case in Table 1. All patients were male, as expected, though more severely affected females have been reported.^13^, ^14^ They had classic features of Arts syndrome, without atypical features like diabetes insipidus, dysmorphic features, or gray matter heterotopia that have been reported previously.^9^, ^10^ Biochemically, many purine metabolites were in the low normal range for these patients; however, two patients did have low hypoxanthine. Of note, some reference ranges for these metabolites include 0. Our patient started SAMe and NR therapy earlier than previously reported patients, at 20 months old compared to after 3 years of age and mid‐childhood.

**TABLE 1:** Comparison of patients with Arts syndrome previously treated with supplementation of S‐adenosylmethionine with or without nicotinamide riboside. PRPP synthetase enzyme activity was unable to be sent due to availability/insurance coverage in patient 1.

Patient	1	2^a^—de Brouwer et al.^4^, ^5^, ^6^	3^a^—de Brouwer et al.^4^, ^5^, ^6^	4—Lenherr et al.^7^
Genotype	c.383A>T (p.Asp128Val)	c.398A>C (p.Gln133Pro)	c.398A>C (p.Gln133Pro)	c.250C>T (p.Arg84Trp)
Inheritance	Mother: affected with hearing loss	Mother: healthy	Mother: healthy	De novo
Age at diagnosis	14 months	11 years	9 years	38 months
Age at follow‐up	28 months	14 years (died at 19 years)	13 years (died at 18 years)	‐
Sex	M	M	M	M
Motor development	Sat unsupported at 10 months, crawled at 14 months, pulled to stand at 18 months, walking with walker at 21 months. Significant regression with hospitalization at 24 months. At 26 months, sitting unsupported, pulling to stand.	‐	‐	Unable to ambulate, loss of head control with recurrent infections
Language development	First words at 26 months	‐	‐	Spoke 10 words at 38 months
Hypotonia	Y	Y	Y	Y
Optic atrophy	Retinal Dystrophy	Y	Y	Y
SNHL	Y—has cochlear implants	Y	Y	Y—has cochlear implants
Ataxia	Y	Y	Y	Y
Peripheral neuropathy	‐	Y—EMG/NCV suggestive of denervation	Y—EMG/NCV suggestive of denervation	Y
Recurrent infections	No recurrent infections, one significant infection	Y—worsening of muscle weakness and respiratory status with each infection	Y—worsening of muscle weakness and respiratory status with each infection	Y
Uric acid (serum)	3.6 (RR 2–6 mg/dL)	0.13 (RR 0.12–0.35 mM)	0.16 (RR 0.12–0.35 mM)	Normal
Uric acid (urine mmol/mol Cr)	799 (RR 350–2500)	490 (RR 200–600)	540 (RR 300–1000)	Normal
Xanthine (urine mmol/mol Cr)	17 (RR < 54)	8 (RR 5–80)	10 (RR 5–80)	Normal
Hypoxanthine (urine mmol/mol Cr)	13 (RR < 65)	<1 (RR 2–55)	<1 (RR 2–55)	Normal
PRPP synthetase activity	‐	0 nmol/mg/h (RR 24–48)	0 nmol/mg/h (RR 24–48)	0.1 nmol/mg/min (RR 0.41–1.46)
Supplementation	40 mg/kg/day SAMe 30 mg/kg/day NR Both starting at 20 months	30 mg/kg/day SAMe starting in mid‐childhood	30 mg/kg/day SAMe starting in mid‐childhood	~28 mg/kg/day SAMe starting at 39 months 300 mg daily NR starting at 43 months
Outcomes	Improved strength and endurance by parental report. Developmental gains despite regression with ICU stay. Limited recurrent infections, however significant cardiac event with upper respiratory infection.	Stabilization of ataxia and hearing impairment. Discontinuation of nighttime BiPAP. Reduced infection frequency and severity. Prior to supplementation: >180 days hospitalized in 84 months. After supplementation: 5 days hospitalized in 33 months.	Stabilization of ataxia and hearing impairment. Discontinuation of nighttime BiPAP. Reduced infection frequency and severity. Prior to supplementation: 82 days hospitalized in 84 months. After supplementation: 0 days hospitalized in 33 months.	Improved interval time between infections from nearly inexistent to 3‐month intervals. Improved strength, speech, ability to play by parental report. Increased ATP, GTP, and NAD(P) levels. Improved T‐cell survival and function.

Abbreviations: Cr, creatinine; EMG, electromyogram; NCV, nerve conduction velocities; NR, nicotinamide riboside; RR, reference range; SAMe, S‐adenosylmethionine; SNHL, sensorineural hearing loss.

^a^ Siblings.

All patients reported clinical stability, improvements in infection burden, or gains in developmental milestones. Notably, the brothers treated with SAMe were able to discontinue nightly BiPAP and had a significant decrease in infection burden.^4^, ^5^, ^8^ The patient treated with SAMe and NR had improved strength, speech, ability to play, and increased time interval between infections.^7^ Lenherr et al. also demonstrated increased ATP, GTP, and NAD(P) levels on therapy, as well as improvement in T‐cell survival and function. In comparison, our patient had reported improvement in strength and endurance with developmental gains after initial treatment. Unfortunately, detailed objective measures were limited by telemedicine. He did not have recurrent infections, with occasional respiratory infections that were reasonable for his age, perhaps influenced by SAMe and NR therapy. This may also be in part due to the infection control measures during the Covid‐19 pandemic. However, while on SAMe and NR, he had one very significant infection with respiratory failure leading to cardiac arrest associated with a significant decline, but then recovered to near baseline and made additional developmental gains within a couple of months. It is not fully clear how the clinical trajectory was impacted by SAMe and NR. There were a few untreated Arts syndrome patients who also reported regained milestones after decline with infection, but they eventually had progressive decline in strength and respiratory function.^2^, ^3^ Longer clinical follow‐up is needed for our patient to monitor for potential infection‐related symptom progression; he has since had no clinical concerns with an additional respiratory infection.

Those who are treated appear to have a different clinical trajectory than the majority of previously reported Arts syndrome patients, who have continued clinical decline overall and often pass away before the age of 5 years.^2^, ^3^, ^4^ The brothers treated with SAMe died at 18–19 years of age.^6^ While our patient and the previously reported patient treated with SAMe and NR are still young, they appear to have stable symptoms and are making developmental gains. Survival beyond early childhood has been reported in a few Arts syndrome patients, including those associated with atypical clinical features.^9^, ^10^

Due to the rarity of this condition, there have not been formal guidelines published for Arts Syndrome. With increased availability and broadened first‐line genetic testing, molecular testing is likely the most common method of diagnosis for Arts syndrome, as biochemical markers are not consistent. *PRPS1* is found on many commercially available genetic panels for hearing loss, intellectual disability, and optic atrophy; however, review of individual panels is recommended. After diagnosis, evaluation for potential associated complications is warranted, particularly with the spectrum of phenotypes associated with PRPS1 deficiency. This can include ophthalmologic evaluation for optic atrophy, retinal dystrophy, and other vision concerns, neurologic evaluation for neuropathy and ataxia, hearing evaluation, and developmental assessment. Biochemical and enzyme studies can be helpful to further characterize the degree of PRPS1 deficiency, as well as differentiate it from PRPS1 superactivity. Clinical experience appears to show the benefit of SAMe and NR in individuals with a severe PRPS1 deficiency phenotype, or Arts syndrome, and we feel that these therapies in Arts syndrome patients should be considered. Clinical data for SAMe and NR in milder phenotypes associated with PRPS1 deficiency are limited. With the case series above, there is ongoing discussion regarding SAMe and NR therapy in a female PRPS1 deficiency patient at our institution who is significantly affected by developmental delay, ataxia, hearing loss, and retinal dystrophy, although additional research is needed to evaluate the effectiveness of SAMe and NR supplementation in PRPS1 deficiency.

Both SAMe and NR were obtained over the counter, as pharmaceutical‐grade products were unavailable. Products obtained were manufactured in a “Good Manufacturing Practices” certified facility compliant with the Food and Drug Administration's manufacturing practice and standards, which includes activities to ensure the identity, purity, and strength of dietary supplements. However, the products themselves were not subject to pharmaceutical regulation of purity, safety, strength, and so on by the Food and Drug Administration. Our team used SAMe from Nature's Craft® and NR from Ultrahealth® due to local convenience and pricing, though there are many brands with similar over‐the‐counter products.

SAMe and NR have been used for a variety of indications with good tolerance. SAMe has been used for major depressive disorder at doses of 200–3200 mg daily that are tolerated well. Side effects were mild and commonly included gastrointestinal symptoms, sweating, vertigo, restlessness, and anxiety.^18^ SAMe was also well tolerated at 800 mg two times daily in a Lesch–Nyhan patient with improved symptoms.^19^ In a study in healthy volunteers, NR dosing of up to 2 g daily was well tolerated without side effects.^20^ In animal toxicology studies, elevations in liver enzymes and lipids were reported at doses >1000 mg/kg/day.^17^ Other forms of B3, including nicotinic acid and nicotinamide, have been associated with flushing; however, this side effect is not commonly reported with nicotinamide riboside.^17^, ^20^ Notably, nicotinic acid and nicotinamide are PRPP‐dependent and would not be an adequate substitute of NR. Our patient has not experienced any of the above side effects thus far and we will continue to monitor clinically. Overall, the side effect profile for SAMe and NR are mild, with more significant symptoms occurring at much higher dosing than was utilized in these patients.

This *PRPS1* c.383A>T variant was previously reported at 70% mosaicism in a 17‐year‐old male with early onset retinal dystrophy, sensorineural hearing loss, axonal and demyelinating neuropathy on electromyogram, hypotonia, and developmental delay.^16^ His phenotype was reported to be closer to CMTX5 without significant infections or related decline, while our patient is more severely affected with an Arts syndrome phenotype. The differences in phenotype may be attributed to mosaicism in the previously reported patient.

In conclusion, this is the second Arts syndrome patient treated with SAMe and NR to our knowledge. He showed overall improvements in strength and endurance, as well as developmental gains. All previously reported Arts syndrome patients treated with SAMe alone, or SAMe and NR, appear to have clinical benefit and both serve as a potential treatment option for these patients, although further studies are warranted.

## AUTHOR CONTRIBUTIONS

Dr. Angela Lee gathered clinical information and prepared the manuscript. Drs. Hoanh Nguyen, Renatta Knox, Margaret Reynolds, and Erin McRoy gathered clinical information and critically revised the manuscript.

## CONFLICT OF INTEREST STATEMENT

The authors declare no conflicts of interest.

## ETHICS STATEMENT

All procedures followed were in accordance with the ethical standards of the responsible committee on human experimentation (institutional and national) and with the Helsinki Declaration of 1975, as revised in 2000. Informed consent was obtained from all patients for being included in the study. This article does not contain any studies with animal subjects performed by any of the authors.

## Data Availability

Additional data are available upon reasonable request of the corresponding author.
